# May-Thurner Syndrome: A Rare, Yet Recognized, Cause of Deep Vein Thrombosis

**DOI:** 10.7759/cureus.66357

**Published:** 2024-08-07

**Authors:** Zaid Al Ghananeem, Amit Deshpande, Vaibhav Sundaresan, Mohammad Abuzenah, Hamza Abuzenah

**Affiliations:** 1 Vascular Surgery, Sheffield Teaching Hospitals NHS Foundation Trust, Sheffield, GBR; 2 Vascular Radiology, Sheffield Teaching Hospitals NHS Foundation Trust, Sheffield, GBR; 3 General Medicine, Sheffield Teaching Hospitals NHS Foundation Trust, Sheffield, GBR; 4 Internal Medicine, Yarmouk University, Irbid, JOR

**Keywords:** may-thurner syndrome, interventional radiology, iliac vein compression, iliac vein stent, endovascular thrombectomy, deep venous thrombosis (dvt)

## Abstract

May-Thurner syndrome (MTS) involves the chronic compression of the left common iliac vein (CIV) by the overlying right common iliac artery (CIA) against the lumbar vertebrae. This compression can result in signs and symptoms of deep vein thrombosis (DVT) affecting the left side. In this case report, we present the clinical details of a 19-year-old patient diagnosed with severe MTS, which manifested as DVT with symptoms of severe thigh pain, redness, and difficulty walking. Additionally, the patient experienced pleuritic chest pain, ultimately diagnosed as pulmonary embolism (PE). Her management involved surgical removal of the thrombus and endovascular stenting of the left CIV. Following her recovery, she progressed favorably, and her follow-up assessment yielded satisfactory results.

## Introduction

May-Thurner syndrome (MTS), also referred to as iliac vein compression syndrome, arises when the left common iliac vein (CIV) becomes compressed by the right common iliac artery (CIA) against the spinal column. This compression often affects the fifth lumbar vertebra [1]. MTS accounts for approximately 2% to 5% of cases involving deep vein thrombosis (DVT) in the lower extremities [1]. In human anatomy, the common iliac veins originate from the fusion of the external iliac veins and internal iliac veins. These left and right common iliac veins converge within the abdomen at the level of the fifth lumbar vertebra, ultimately forming the inferior vena cava. Their primary function is to drain blood from the pelvis and lower limbs. Throughout their course, the common iliac veins run alongside the common iliac arteries.

While both the left and right CIVs lie deep to the right CIA, there is an important anatomical difference. The left CIV follows a more transverse course and is susceptible to compression, whereas the right CIV ascends more vertically and is not similarly predisposed. This variant anatomy contributes to the increased occurrence and prevalence of left iliofemoral or left iliac vein thrombosis [2]. The exact incidence and prevalence remain uncertain because most patients are clinically asymptomatic [3]. Venous collaterals can develop to maintain blood flow, particularly when the obstruction is not severe [4].

When diagnosing MTS, several approaches come into play. First, during the workup for DVT, clinicians may identify MTS. Additionally, suspicion of MTS based on clinical presentation and risk factors prompts further investigation. While color Doppler ultrasound is essential for evaluating suspected DVT, it alone is inadequate for confirming MTS diagnosis. For that, cross-sectional iliocaval imaging is necessary [5,6]. Standard imaging techniques for MTS encompass magnetic resonance venography (MRV) and intravascular ultrasound (IVUS) [6]. This case report presents a severe DVT attributed to MTS, with diagnosis established using the previously mentioned imaging techniques.

## Case presentation

**Figure 1:**
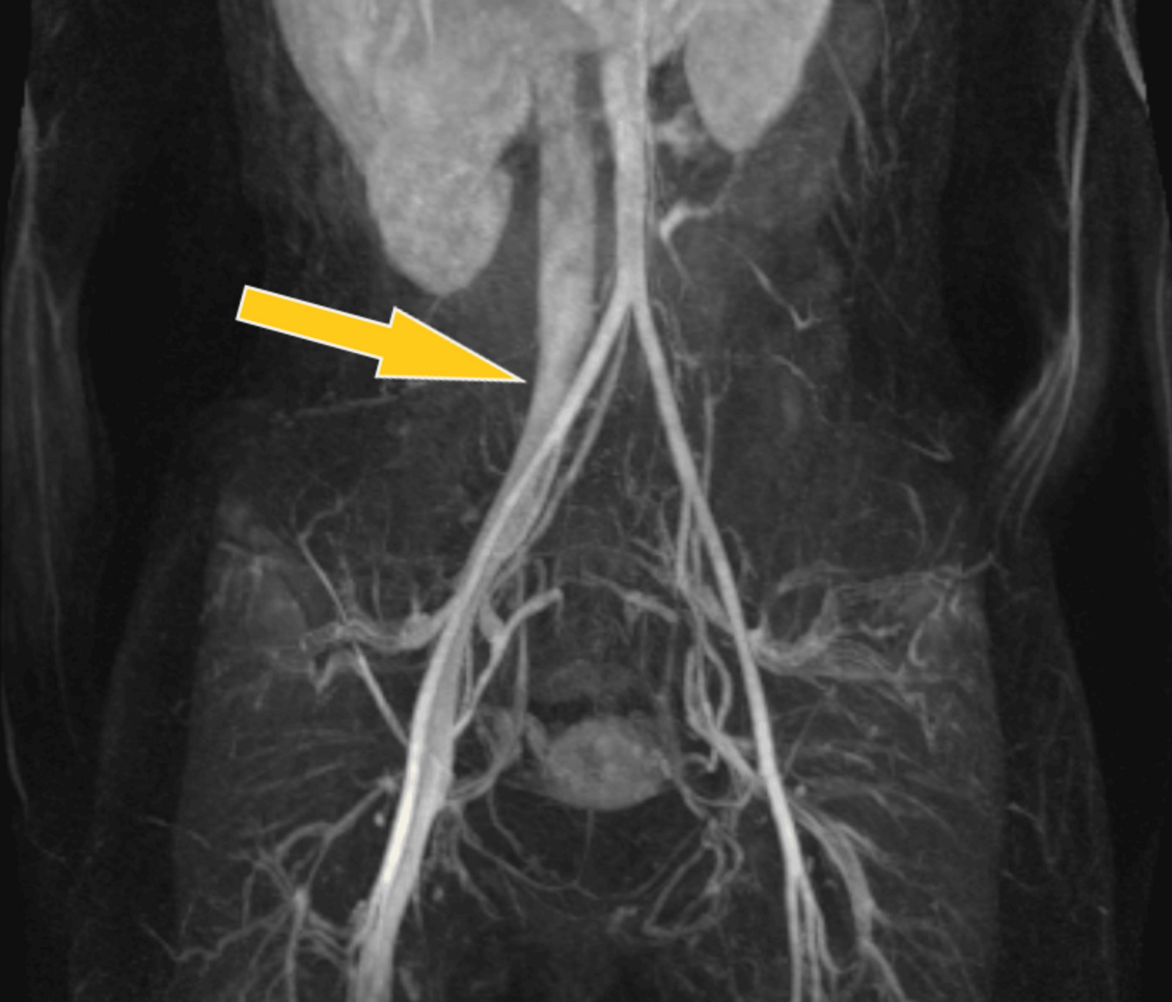
MR venogram of the iliac and femoral veins In this MR venogram image, the patent right iliofemoral veins are highlighted by the yellow arrow. However, it’s important to observe that there is no left iliofemoral venous filling due to the complete occlusion of the left iliofemoral veins.

We present the case of a 19-year-old, female university student who is a non-smoker with an unremarkable medical history. She sought care at Sheffield Teaching Hospitals' (STH) emergency department due to severe left thigh swelling, redness, pain, and an inability to walk. Additionally, she experienced pleuritic chest pain, which was later diagnosed as pulmonary embolism (PE) following elevated D-dimer levels and computed tomography pulmonary angiogram (CTPA) scan. Aside from oral contraceptive pills (OCP), she was not on any other medications. The patient denied recent travel, surgery, trauma, or prior thromboembolic events. She had no history of tobacco, alcohol, or illicit drug use, and her family had no clotting disorders. Ultrasound (US) scan showed DVT in the left external iliac vein (EIV), common femoral vein (CFV), and profunda femoral vein. The MRV scan done on admission showed complete occlusion of the left iliofemoral venous system (Figure 1).

Upon initial laboratory assessment, the complete blood count revealed a hemoglobin level of 133 g/L, a white blood cell count of 10.4 x 109/L, and a platelet count of 248 x 109/L. Kidney function tests were unremarkable except for mildly low urea. Coagulation parameters, including prothrombin time (PT), activated partial thromboplastin time (APTT), and fibrinogen were all within normal ranges. However, the D-dimer level was markedly elevated, exceeding 50,000 µg/L. Additionally, the C-reactive protein level was measured at 112 mg/L (Table 1).

**Table 1:** Initial laboratory results upon admission The results reveal a normal coagulation profile, except for a high D-dimer level. There is no evidence of infection or renal dysfunction, which is important for excluding other potential causes of the patient’s symptoms.

Laboratory test	Result	Normal range
Hemoglobin	133	110 – 147 (g/L)
White Blood Cell Count	10.4	3.5 – 9.5 (x 10^9^/L)
Platelet Count	248	150 – 400 (x 10^9^/L)
Urea	1.8	2.5 – 7.8 (mmol/L)
Creatinine	53	44 – 80 (umol/L)
C-Reactive Protein	112	0 – 5 (mg/L)
Prothrombin Time (PT)	9.8	9.2 – 11.2 (seconds)
Activated Partial Thromboplastin Time (APTT)	21.4	20.6 – 26.6 (seconds)
Fibrinogen	2.9	2.0 – 4.0 (g/L)
D-Dimer	>50000	0 – 500 (ug/L)

**Figure 2:**
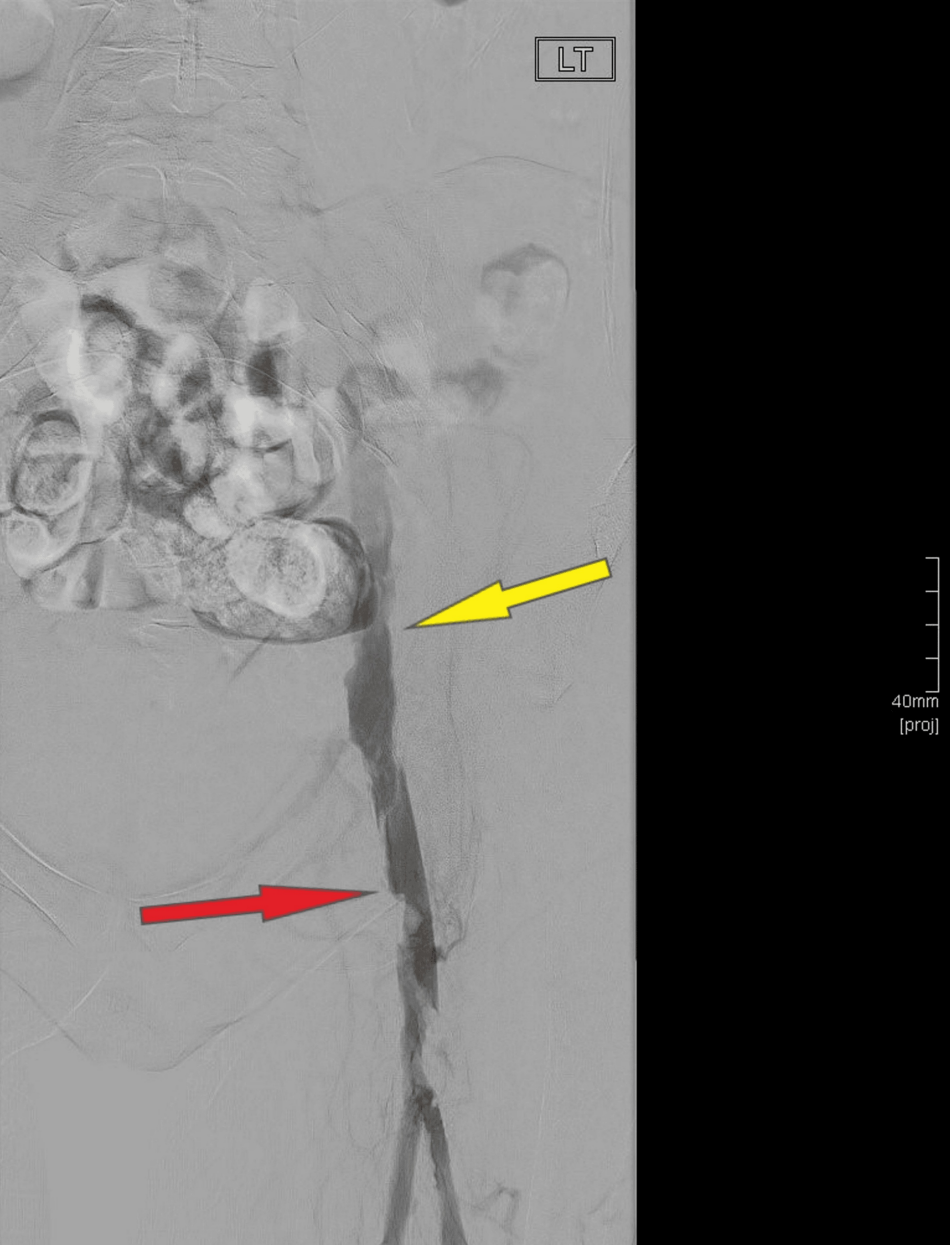
Left lower limb venography This venogram image was taken in the angioplasty suite after completing the thrombectomy. It reveals a patent left common iliac vein (indicated by the yellow arrow) and left common femoral vein (highlighted by the red arrow), along with minor residual thrombi.

Following admission, the patient was started on continuous intravenous (IV) heparin infusion at a dose of 800 international units (IU) with an infusion rate of 0.8 mL per hour. Subsequent adjustments were made to the infusion rate based on monitoring of the APTT ratio. Afterward, she underwent an urgent venous thrombectomy of the left lower limb due to acute left iliofemoral DVT. The procedure was performed under local anesthesia using a combination of 10 ml 1% lidocaine and bupivacaine. Pre-procedure duplex ultrasound confirmed an occluded left popliteal vein, which had progressed since the MRV scan. Access was achieved using a microneedle-microwire combination guided by ultrasound through the left popliteal vein. An 8 French gauge (Fr) sheath was introduced into the occluded popliteal vein using a micropuncture set. The initial venogram revealed occluded left popliteal and femoral veins. Subsequently, suction thrombectomy was performed using the Cat 7 Lightning Penumbra System (Penumbra, Inc., California, US) successfully removing a large amount of clot from the left popliteal vein, superficial femoral vein (SFV), profunda vein, and iliac veins. The final venogram demonstrated patent femoral and iliac veins (Figure 2), although some residual thrombi remained in the left EIV and CFV. Following this successful thrombectomy, the patient continued to receive IV heparin 800 IU at a rate of 0.8 mL per hour and was admitted to the high dependency unit (HDU) for close monitoring.

The following day, the patient underwent the second stage of treatment for MTS, which involved left iliac vein angioplasty and stenting under general anesthesia (GA). Prior to the procedure, a venogram revealed patent left femoral and iliac veins, along with significant stenosis in the left CIV. The stenosis was confirmed using IVUS. Pre-dilation was performed using a 6 mm Mustang balloon (Boston Scientific, Marlborough, Massachusetts, US) followed by angioplasty with a 14 mm Atlas Gold balloon. Then, an Abre Medtronic venous self-expanding stent (Medtronic plc, Minneapolis, Minnesota, US) was inserted from the left CIV to the left EIV. Post-dilation measurements showed a 14 mm diameter in the left CIV and a 12 mm diameter in the left EIV. Overall, the angiography outcome was satisfactory, with good contrast flow through the left femoral and iliac veins (Video 1).

**Video 1:** Angiography of the left femoral and iliac veins The angiography reveals that the left femoral and iliac veins are patent, with satisfactory venous filling and the stent properly inserted.

After undergoing the two procedures, the patient was stepped down to ward-level care and was switched from intravenous heparin to a therapeutic dose of low molecular weight heparin (LMWH). Over the next 48 hours, her symptoms began to improve, and she felt better. Subsequently, she was discharged with a prescription for apixaban (5 mg twice daily). Two weeks after discharge, a follow-up ultrasound scan showed no evidence of DVT in her left EIV, CFV, or popliteal veins. Clinically, she was progressing well, experiencing significant symptom improvement.

## Discussion

The diagnostic and management challenges associated with MTS, especially in the presence of a large, left-sided DVT, highlight the intricate interplay between uncommon anatomical variations and more common vascular conditions. While angioplasty and stenting remain the primary approach for most MTS patients, there exists an alternative, less frequently employed procedure - thrombectomy, often followed by stent placement [5,6]. For asymptomatic patients, treatment options include blood thinners and the use of supportive measures such as compression stockings [5,6].

The thrombectomy approach, which is often accompanied by stent placement, is primarily recommended for massive DVT cases where complete obstruction of the affected venous system occurs. In such situations, stenting alone may prove insufficient. Consequently, urgent revascularization of the venous system becomes necessary to avert worsening and potentially catastrophic outcomes. Additionally, this approach is reserved for patients who cannot undergo standard venous lysis therapy due to contraindications [7]. These contraindications encompass conditions like active bleeding, vascular malformations, aneurysms, and cerebral infarctions [8].

The use of systemic anticoagulation alone is not considered the standard of care for these patients. Studies have demonstrated that relying solely on anticoagulation may result in recurrent DVT [5,6]. Consequently, endovascular therapy becomes indispensable for the safe management of these patients. Addressing DVT resulting from MTS requires a refined approach that addresses both the acute thrombotic event and the underlying venous compression [9].

When exploring potential differential diagnoses for DVT, it’s essential to consider a range of conditions, including cellulitis, lymphedema, and a ruptured Baker’s cyst [9,10]. However, when faced with left-sided DVT that lacks apparent risk factors, the differential diagnosis becomes narrower, shining a spotlight on the significance of MTS. The clinical similarities between these conditions and MTS-associated DVT can sometimes obscure the diagnostic path, underscoring the need for heightened clinical suspicion and judicious use of diagnostic imaging [9,10].

Compared to males, females exhibit a higher incidence of MTS [11]. The prevailing standard of care for symptomatic MTS entails a dual approach: immediate anticoagulation followed by subsequent endovascular intervention. Endovascular management typically includes venography, IVUS-guided angioplasty, and stenting of the left CIV [5]. While PE remains a concern whenever DVT is present, intriguingly, research has revealed that the incidence of PEs in patients with MTS is significantly lower compared to the typical PE risk associated with DVT. This phenomenon likely stems from the unique venous compression seen in MTS [12]. Hence, the placement of an inferior vena cava filter is not advisable for these patients.

While anticoagulation is essential for preventing additional thrombus formation, it has a limited impact on addressing the underlying venous obstruction [9]. Thrombectomy and stenting serve a dual purpose: they address acute symptoms while also working to prevent long-term complications by restoring venous flow [13]. Patients typically initiate anticoagulation therapy to prevent recurrence and post-stent thrombosis. The extension of treatment is guided by underlying thrombotic and bleeding risk factors. Although there are limited studies on this topic, warfarin remains the anticoagulant of choice in most literature. While evidence for non-vitamin K oral anticoagulants is scarce, there are case reports documenting the successful use of direct oral anticoagulants (DOACs) [14].

The patient's initial presentation with a chief complaint of left thigh pain and swelling, along with a history of OCP use, could have been attributed to provoked DVT due to OCPs. While this is considered a common cause, the severity of the clinical presentation and the patient’s age prompted further investigation. The appropriate radiographic imaging revealed a rare structural cause of DVT in the form of complete occlusion of the main veins in the left lower limb. Given this finding, prompt intervention became imperative. This case serves as an illustrative example of the importance of not overlooking rare DVT causes, which can have fatal or disabling consequences if left untreated. Additionally, it underscores the role of surgical management in cases where medical treatment alone may not suffice.

## Conclusions

This case report highlights the safety and effectiveness of thrombectomy, angioplasty, and stenting in managing acute occlusive DVT secondary to MTS. Following the patient’s presentation to the emergency department and appropriate management, her occlusion was successfully treated using endovascular stenting and thrombectomy. Beyond addressing the diagnostic and therapeutic challenges associated with MTS, this case underscores the critical importance of considering anatomical factors in vascular medicine. While MTS can sometimes be asymptomatic, it can also lead to severe and life-threatening complications, as illustrated by this case. Early recognition and timely treatment are crucial. Ongoing research aims to further improve patient care.
